# *Lavandula austroapennina*: Assessment of the Antiviral Activity of Lipophilic Extracts from Its Organs

**DOI:** 10.3390/v15081648

**Published:** 2023-07-28

**Authors:** Annalisa Chianese, Claudia Gravina, Maria Vittoria Morone, Annalisa Ambrosino, Marialuisa Formato, Francesca Palma, Francesco Foglia, Bianca Maria Nastri, Carla Zannella, Assunta Esposito, Anna De Filippis, Simona Piccolella, Massimiliano Galdiero, Severina Pacifico

**Affiliations:** 1 Department of Experimental Medicine, University of Campania “Luigi Vanvitelli”, 80138 Naples, Italy; annalisa.chianese@unicampania.it (A.C.); mariavittoria.morone@unicampania.it (M.V.M.); annalisa.ambrosino@unicampania.it (A.A.); francesca.palma@unicampania.it (F.P.); francesco.foglia@unicampania.it (F.F.); biancamaria.nastri@unicampania.it (B.M.N.); carla.zannella@unicampania.it (C.Z.); anna.defilippis@unicampania.it (A.D.F.); 2 Department of Environmental, Biological and Pharmaceutical Sciences and Technologies, University of Campania “Luigi Vanvitelli”, 81100 Caserta, Italy; claudia.gravina@unicampania.it (C.G.); marialuisa.formato@unicampania.it (M.F.); assunta.esposito@unicampania.it (A.E.); simona.piccolella@unicampania.it (S.P.)

**Keywords:** *Lavandula austroapennina*, Lamiaceae, UHPLC Q*q*TOF-MS/MS analysis, fatty acids, triterpenes, antiviral activity, enveloped virus

## Abstract

In a framework aimed at the recovery and enhancement of medicinal plants endemic to the territory of the Cilento and Vallo di Diano National Park, *Lavandula austroapennina* N.G. Passal., Tundis and Upson has aroused interest. An insight into the chemical composition of the corolla, calyx, leaf, stem, and root organs was carried out following ultrasound-assisted maceration in *n*-hexane. The obtained lipophilic extracts were explored using ultra-high-performance chromatography coupled to high-resolution mass spectrometry (UHPLC-ESI-QqTOF-MS/MS). The extracts from the different organs varied in their relative content of fatty acids, ursanes, and oleanane-type triterpenes. In particular, the oleanolic acid content appeared to increase in the order of corolla < leaf < stem. An MTT assay was performed to verify the possible cytotoxicity of the organ extracts of *L. austroapennina* at a concentration ranging from 12.5 to 400 µg/mL on the Vero CCL-81 cell line. Antiviral activity against herpes simplex virus type 1 (HSV-1), alpha human coronavirus 229E (HCoV-229E), and poliovirus type 1 (PV-1) was evaluated via a plaque reduction assay in the same cellular model. All the extracts did not show cytotoxic effects after 2 and 24 h exposure times, and the antiviral efficacy was particularly important for the stem extract, capable of completely inhibiting the tested viruses at low doses. The antiviral activity in a non-enveloped virus PV-1 allowed the assertion that the extracts from the organs of *L. austroapennina*, and especially the stem extract, interfered directly with the viral envelope. This study underlines how much knowledge of a territory’s medicinal plant heritage is a harbinger of promising discoveries in the health field.

## 1. Introduction

Emerging and re-emerging viral infections remain a large-scale challenge all over the world. The emergence arises from rapidly evolving pathogens and changes in the environment, as well as in a host providing such agents with suitable new ecological niches. The last decades have recorded unprecedented pandemic outbreaks: severe acute respiratory syndrome (SARS) in 2003, H1N1 “swine” influenza (2009), Middle East respiratory syndrome (MERS) in 2012, chikungunya (2014), and Zika (2015), as well as the last pandemic explosion due to SARS-CoV-2 (2020 to the present). Alongside these infections, many past emerging infectious agents stably co-exist with humans, and it is demonstrated by the persistence of endogenous retroviruses in human DNA [1] and by herpesviruses that are able to latently infect.

Nowadays, several life-threatening viruses, including human immunodeficiency virus (HIV); hepatitis virus subtypes A, B, and C (HAV, HBV, and HCV); herpes simplex virus (HSV); and SARS-CoV-2, have become some of the greatest global health challenges due to uncontrolled morbidity and mortality rates [2,3,4,5]. It is certain that the variability in viruses requires antiviral drugs that are able to interfere with many viral targets [6,7].

In this context, while antiviral synthetic drugs have been developed, natural products have gained a renewed interest, thanks to their ability to avoid side effects and the low production costs [8].

Indeed, there is a growing awareness that several specialized plant metabolites offer a wide variety of antiviral therapeutic compounds [9,10], being capable of decreasing viral lifecycle processes: cell surface adhesion, cell entry, viral genome replication, viral protein expression, and the assembly and release of viral particles. Medicinal and aromatic plants (MAPs) are historically of interest for their beneficial and therapeutic properties [11]. The World Health Organization (WHO) aims to strengthen the role of traditional medicine by promoting the use of medicinal plants in the health systems of its countries [12]. This has led to a renewed use of MAPs in developed countries, while on the contrary, MAPs are the first therapeutic strategy in low- and medium-developed countries [13]. Among MAPs, species from the *Lavandula* genus (Lamiaceae)—such as *L. angustifolia* Mill., *L. latifolia* Medik., *L. pedunculata* (Mill.) Cav., *L. stoechas* L., and *L. × intermedia* Emeric ex Loisel—are broadly cultivated worldwide and investigated for their antioxidant, anti-inflammatory, antidepressant, spasmolytic, anticholinesterases, and antimicrobial efficacy [14,15,16]. In particular, a lavender essential oil was proposed as an antiviral agent against the avian influenza H5N1 virus [17], while lavender syrup was found to be effective on COVID-19-induced olfactory dysfunction [18].

Therefore, the screening of the yet un-investigated medicinal plants could open up new scenarios for the future treatment of viral infections, also based on their ability to combine high safety and efficacy.

Within this framework and as part of a project aimed at promoting the endemic species of Southern Italy [8,19,20], in particular of the Campania Region, through also differently exploiting their organs, *Lavandula austroapennina* (*L. austroapennina*) N.G. Passal., Tundis and Upson—which was morphologically and genetically identified as a disjunct population of *L. angustifolia* subsp. *angustifolia* [21,22,23]—has been investigated. To this purpose, *L. austroapennina* was dissected in its organs (corolla, calyx, leaves, stem, and roots), and each one was further subjected to extraction in *n*-hexane. Thus, the extracts, chemically analyzed using UHPLC-ESI-Q*q*TOF-MS/MS tools, underwent a dose-response cytotoxicity evaluation on the epithelial Vero cell line, and an antiviral screening against HSV-1, HCoV-229E, and PV-1 was performed.

## 2. Materials and Methods

### 2.1. Plant Material and Extraction

Plants of *L. austroapennina* were collected in July 2022 in the wild on Mt. Cervati (Sanza Municipality, 40°15′19.6″ N 15°28′42.8″ E, 11,801,250 m a.s.l.) in the National Park of Cilento, Vallo di Diano and Alburni (Southern Italy). A voucher specimen of the plant (N.0131), taxonomically identified following Pignatti et al. [24,25], was deposited in the *Herbarium Austroitalicum* (IT, acronym following Thiers 2023) of the University of Campania Luigi Vanvitelli (Caserta, Italy). At the collection site, each plant was dissected into the organs of the corolla, calyx, leaf, stem, and root, which were stored in liquid nitrogen and transferred to the laboratory for the cell-based assays. The plant material underwent freeze-drying using the FTS System Flex-DryTM instrument (SP Scientific, Stone Ridge, NY, USA), following pulverization (Knife Mill PULVERISETTE 11, Buch & Holm, Herlev, Denmark). A total of 2.0 g of each sample underwent ultrasound-assisted maceration (UAM; Branson Ultrasonics^TM^ Bransonic^TM^ M3800-E, Danbury, CT, USA) using *n*-hexane as an extractive solvent. The plant organ/solvent ratio was 1:20 (g plant organ: mL solvent); three UAM cycles were carried out (30 min for each) away from light. At the end of each cycle, the samples were filtrated, and the extracts obtained were dried using a rotary evaporator (Heidolph Hei-VAP Advanyage, Schwabach, Germany).

The apolar extract of each organ was chemically investigated using UHPLC-ESI-QqTOF-MS/MS.

### 2.2. UHPLC-ESI-QqTOF-MS/MS Analyses

The apolar extracts from *L. austroapennina* organs (10 mg/mL) were analyzed with a NEXERA UHPLC system (Shimadzu, Tokyo, Japan) equipped with a Luna^®^ Omega C-18 column (50 × 2.1 mm i.d., 1.6 μm particle size). The mobile phase consisted of water (solvent A) and acetonitrile (solvent B), both acidified with formic acid (0.1% *v*/*v*). A linear gradient was used with an increasing solvent B percentage: 0–2.50 min, 50% B; 2.5–9.0 min, 50%→95% B; 9.0–10.0 min, held at 95%. Then, the mobile phase composition was allowed to re-equilibrate for 2 min. The flow rate and the injection volume were 0.5 mL/min and 2.0 µL, respectively. High-resolution mass spectrometry (HR-MS) analyses were carried out using the AB SCIEX Triple TOF^®^ 4600 mass spectrometer (AB Sciex, Concord, ON, Canada), equipped with a DuoSpray^TM^ ion source (AB Sciex, Concord, ON, Canada) operating in the negative ElectroSpray (ESI) mode. The APCI probe was used for automated mass calibration in all scan functions using the Calibrant Delivery System (CDS). A full scan Time-of-Flight (TOF) survey (accumulation time 100 ms, 150–1500 Da) and eight information-dependent acquisition MS/MS scans (accumulation time 50 ms, 100–1350 Da) were acquired using the following parameters: curtain gas at 35 psi, nebulizer and heated gases at 60 psi, ion spray voltage of −4500 V, interface heater temperature at 600 °C, declustering potential (DP) of −80 V, collision energy (CE) of 40 V, and CE spread of 20 V. The compounds were identified mainly through the study of their tandem mass spectrometry (TOF-MS/MS; AB Sciex, Concord, ON, Canada) fragmentation patterns and the comparison with the literature data whenever possible. The instrument was controlled with Analyst^®^ TF 1.7 software (AB Sciex, Concord, ON, Canada), whereas MS data were processed with PeakView^®^ software version 2.2 (AB Sciex, Concord, ON, Canada).

### 2.3. Cell Lines and Cytotoxicity Test

African green monkey kidney epithelial cell line (Vero CCL-81) was purchased from American Type Culture Collection (ATCC, Manassas, VA, USA). Vero cells were grown in Dulbecco’s Modified Eagle Medium (DMEM) with 4.5 g/L of glucose (Microtech, Naples, Italy) supplemented with 10% heat-inactivated fetal bovine serum (FBS, Microgem, Naples, Italy), 2 mM of L-glutamine (Microtech), and 100 IU/mL of penicillin–streptomycin solution (Himedia, Naples, Italy) and maintained at 37 °C in a humidified atmosphere with 5% CO_2_. The cytotoxic effect of the five extracts (all dissolved in DMSO 100%) was evaluated via the 3-[4,5-dimethylthiazol-2-yl]-2,5 diphenyl tetrazolium bromide (MTT) assay (Sigma-Aldrich, St. Louis, MO, USA) based on the manufacturer’s instruction. Basically, 2 × 10^4^ cells/well were seeded in 96-well plates and incubated overnight (O/N) at 37 °C in a humidified atmosphere. Cells were treated with different concentrations (400, 200, 100, 50, 25, and 12.5 µg/mL) of each extract for 2 and 24 h. Then, cell monolayers were treated with MTT solution (0.5 mg/mL) for 3 h in the dark. The amount of soluble formazan was evaluated by recording the absorbance (Abs) at the wavelength of 570 nm with a TECAN M-200 reader (Tecan, Männedorf, Switzerland). The cell survival was calculated by applying the following formula:

$$\%\;\mathrm{of}\;\mathrm{cell}\;\mathrm{viability}=\left(\frac{absorbance\;of\;treated\;samples}{absorbance\;of\;untreated\;samples}\right)$$

### 2.4. Virus and Antiviral Activity

HSV-1 strain SC16, HCoV-229E (ATCC VR-740), and poliovirus type 1 (PV-1) strain Chat (ATCC VR-1562) were propagated on Vero CCL-81 cells.

**Figure 1:**
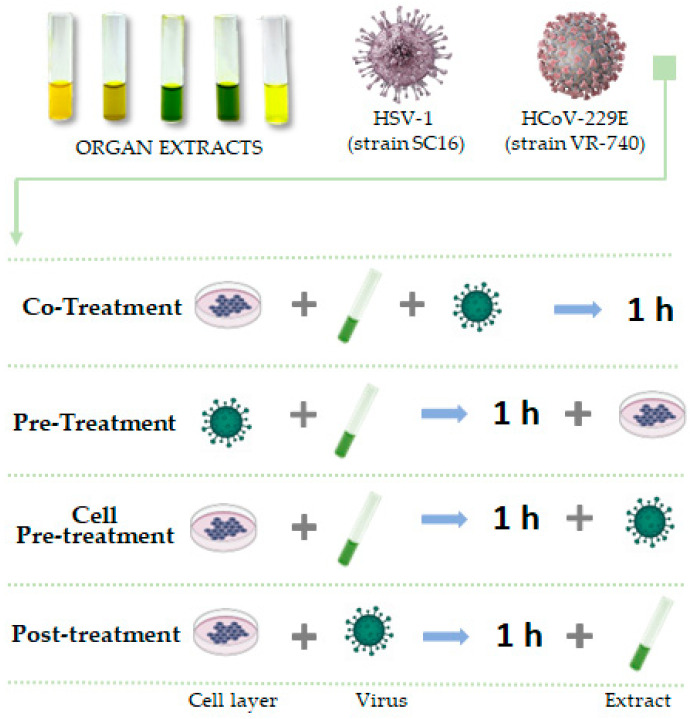
Treatment design of antiviral assays.

Plaque reduction assay was performed to determine the antiviral activity of each extract. Vero cells (1.3 × 10^5^ cells/well) were seeded in 24-well plates and incubated O/N. The monolayer was treated with different dilutions of the compounds, ranging from 200 to 0.8 µg/mL, and infected with viruses at 0.01 multiplicity of infection (MOI). Following a 1 h adsorption at 37 °C, the monolayer was washed with acidic citrate buffer (pH 3.0) to inactivate non-penetrated viruses and incubated for 24–48 h at 37 °C in DMEM supplemented with 3% carboxymethylcellulose (CMC). Finally, cells were fixed with 4% formaldehyde and stained with 0.5% crystal violet for the detection of plaques. A set of treatments (Figure 1) was performed in order to evaluate if extracts had an antiviral potential and in which stage of the viral infection they could act:

- Co-treatment assay: the monolayer was treated and infected simultaneously with extracts and viruses for 1 h;
- Pre-treatment assay: Extracts were previously incubated for 1 h with viruses at 0.1 MOI at 37 °C. Cells were then infected with dilutions of the viral mixture;
- Cell pre-treatment: cell monolayer was pre-treated with compounds for 1 h and then infected with virus;
- Post-treatment: cell monolayer was first infected with virus, then treated with extracts.

The percentage of viral inhibition was measured using the formula:

$$\%\;\mathrm{viral}\;\mathrm{inhibition}=\left(1-\frac{number\;of\;plaques\;in\;treated\;cells}{number\;of\;plaques\;in\;negative\;control}\right)\times 100$$

### 2.5. Statistical Analysis

Cytotoxicity and antiviral tests were performed in triplicate and expressed as mean ± Standard Deviation (SD) calculated with GraphPad Prism (version 5). Statistical differences were evaluated via one-way ANOVA followed with a Dunnett test; a value of *p* ≤ 0.05 was considered significant.

## 3. Results

### 3.1. Lipid Profile of the Different L. austroapennina Organs

UHPLC-ESI-Qq-TOF-MS/MS analysis was carried out to unravel the chemical composition of the *n*-hexane extracts from the corolla, calyx, stem, leaf, and root of *L. austroapennina*. TOF-MS and TOF-MS/MS data of tentatively identified compounds are reported in Table 1.

Fatty acids and triterpenes were the main compounds in all the extracts, while root extract also accounted for a minor diterpene component. In fact, compounds **1**, **2**, **5**, and **7** were likely rosmaridiphenol, rosmanol, rosmaquinone B, and carnosic acid, respectively (Figure S1). These compounds are rarely reported in *Lavandula* species [26], while appearing as main constituents of volatile mixtures from other species belonging to the Lamiaceae family [27]. Indeed, pimarane diterpenes were previously isolated from the alcoholic extract of *L. multifida* aerial parts [26], but no evidence of their occurrence are in the investigated samples.

**Figure 2:**
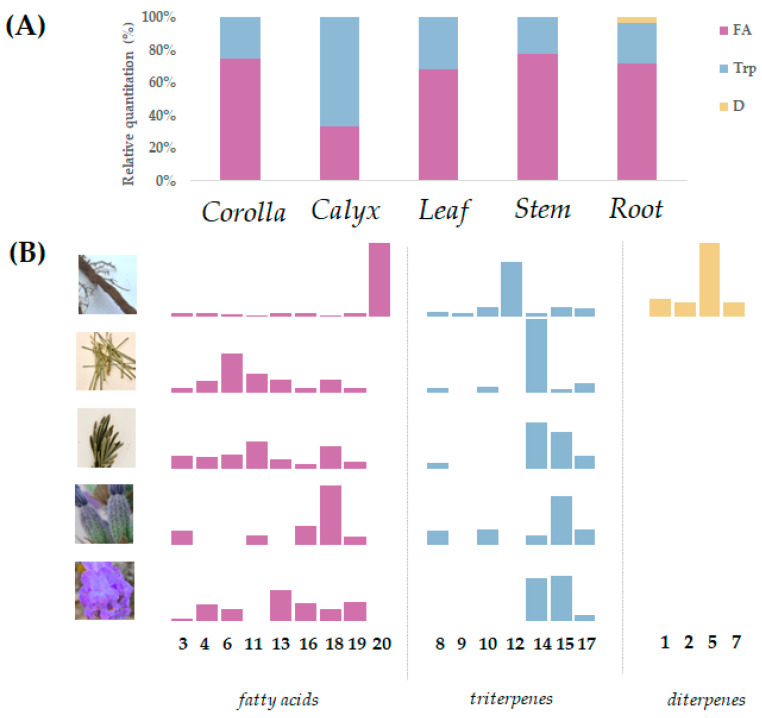
Relative quantitation of identified metabolites in apolar extracts from *L. austroapennina* organs. (**A**) Stacked column chart, comparing the percentage of each metabolic class against the total of all identified classes (FA = fatty acids; Trp = triterpenes; D = diterpenes); (**B**) conditional formatting highlights the trend in terms of occurrence of the single compound in the different investigated organs.

Compounds **3**, **4**, **6**, **11**, **13**, **16**, **18**, and **19** were fatty acids (Figure S2), distinguishable based on an unsaturation degree and/or hydroxylation/oxygenation pattern. Fatty acids were the most abundant compounds in the corolla, stem, radix, and leaf (Figure 2).

**Figure 3:**
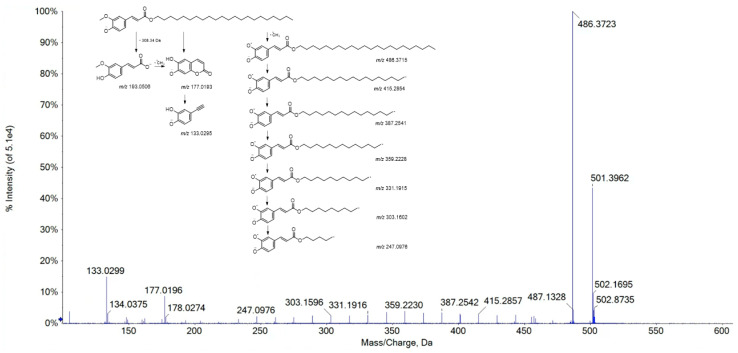
TOF-MS/MS spectrum of compound **20**. The proposed fragmentation pattern is also reported, with theoretical *m*/*z* values.

The saturated palmitic acid (**18**) with the deprotonated molecular ion at *m*/*z* 255.2334 was tentatively identified, beyond the monounsaturated oleic acid (**19**) with [M-H]^−^ at *m*/*z* 281.2489. These compounds, together with compounds **13** ([M-H]^−^ ion at *m*/*z* 277.2178) and **16** ([M-H]^−^ ion at *m*/*z* 279.2334)—likely linolenic and linoleic acid, respectively—were previously found as constituents of apolar extracts of the stem and leaf of *L. officinalis* [28]. TOF-MS/MS spectra of compounds **3** and **4** allowed dihydroxy- and/or oxo-octadecadienoic acids to be putatively identified, based on the relative recognition of β-scission and allyl scission pathways, while compound **6** belonged to octadecatrienoic acids. The deprotonated molecular ion of compound **11** at *m*/*z* 297.2446 was in line with a hydroxyoctadec-12-enoic acid occurrence. Compound **20**, which was only in the root extract, was tentatively identified as docosyl ferulate (Figure 3).

In fact, the [M-H]^−^ ion at *m*/*z* 501.3962 provided—beyond the fragment ion at *m*/*z* 486.3723, due to the methyl radical loss—the fragment ions at *m*/*z* 177.0196 and 133.0299, deriving from feruloyl moiety, as well as the ion at *m*/*z* 193.0508 (corresponding to ferulate ion, although this latter had an intensity lower than 1%). Other fragment ions were detectable and were, according to homolytic cleavages, at different levels on the hydrocarbon chain.

The other compounds were tentatively identified as pentacyclic triterpenes, mainly based on ursane and oleanane skeletons, with different hydroxylation patterns. In particular, the compounds **14** and **15** (Figure S3), sharing the deprotonated molecular ion at *m*/*z* 455.3539(6), were identified as oleanolic acid and ursolic acid, respectively, based on the comparison of their relative retention time with those of pure reference compounds. These compounds were abundant in all the investigated organs and, comparably to a previous observation of *L. pubescens*, oleanolic acid appeared to be highly present in the stems and leaves [29]. The highest relative levels of ursolic acid were detected in the corolla organ, which was listed as the triterpene constituents of *L. angustifolia* flos [30] and leaves [31]. Ursolic-acid-enriched extracts from *L. lusieri* (Rozeira) Riv.- Mart were antimicrobial towards both Gram-positive and -negative bacteria [32]. Compound **17** (Figure S3), with the deprotonated molecular ion at *m*/*z* 453.3381, was a dehydro-derivative of ursolic acid, putatively ursinic acid (3-oxours-12-en-28-oic acid) [33], while compound **8** was likely an ursolic acid trihydroxy derivative. In this context, tormentic acid was reported as a constituent of the *Lavandula* species [34]. Indeed, the TOF-MS/MS spectrum of compound **8** showed—beyond its main fragment ions at *m*/*z* 469.3331 (−18 Da), 443.3543 (−44 Da), and 425.3430 [−(18 + 44) Da]—the fragment ion at *m*/*z* 409.3113, likely due to the loss of vicinal diol function in A-ring and water from the deprotonated molecular ion [35] (Figure S4). Compounds **9** and **10** were tentatively identified as dioxo-hydroxyurs-12-ene-28-oic acid and 3-oxo-hydroxyurs-12-en-28-oic acid, respectively (Figure S3). This latter compound was previously reported as a triterpenoid constituent of the roots of the *Lavandula stoechas* ssp. *stoechas* [36]. The TOF-MS/MS spectra of both compounds highlighted the occurrence of water and carbon dioxide losses, which was also detectable for compound **12** (Figure S3), which was likely a dehydro-derivative of compound **10**.

The relative quantization, which was calculated taking into account the sum of the individual metabolites by metabolic class identified, highlights in particular that the calyx extract differed in the higher content of triterpenes (Figure 2). A metabolite-specific contribution appears to be evidenced when the variation in the relative amount of each compound was considered. Palmitic acid (**18**) was the most abundant fatty acid in the calyx fraction, whereas its content decreased in the leaf extract and, more markedly, in the stem and root fractions. Corolla and calyx extracts also contained a comparable amount of linoleic acid (**16**), while corolla was distinguishable for its content in linolenic acid (**13**) and the appreciable oleic acid (**19**) abundance. Similarly, a differentiation of the triterpene constituents showed that ursolic acid (**15**) was highly contained in the corolla, and calyx decreased in leaf extract, and more markedly, in stem and root mixtures, whereas its constitutional isomer—oleanolic acid **(14**)—increases relatively from the calyx to the leaf, reaching the highest percentage content in stem.

### 3.2. Cytotoxicity Screening of Apolar Extracts from L. austroapennina Organs

**Figure 4:**
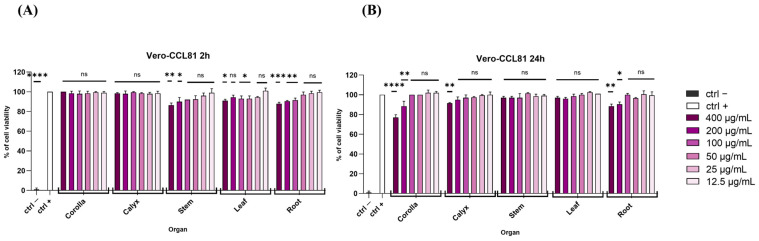
Cell viability evaluation. The viability of Vero cells was analyzed after (**A**) 2 h and (**B**) 24 h with the extract exposition. Dimethyl sulfoxide (DMSO) 100% was used as negative control (ctrl−), while non-treated cells were used as positive control (ctrl+). **** *p* < 0.0001; *** *p* = 0.0001; ** *p* = 0.0011; * *p* = 0.01; ns: non-significant.

Vero cell lines were preliminarily used for carrying out the in vitro evaluation of *L. austroapennina* organs cytotoxicity through an MTT test. Vero cells were used due to their susceptibility to several types of viruses as cell substrates for human vaccines [37] due to the genomic basis of a non-tumorigenic permanent cell line [38]. An MTT test is able to measure the capacity of the mitochondrial dehydrogenases to reduce the tetrazolium ring of MTT, which is yellow-coloured, generating a chromogenic compound, the purple formazan. As shown in Figure 4A,B, fixing a threshold line at 70%, none of the concentrations of each *L. austroapennina* organ extract affected cell viability significantly at both time points. Only the corolla, at 400 and 200 µg/mL, exhibited a slight toxicity after 24 h of incubation.

### 3.3. Antiviral Activity of Apolar Extracts from L. austroapennina Organs against HSV-1 and HCoV-229E

In order to explore the antiviral potential and elucidate the most likely mechanism of action of *L. austroapennina* apolar extracts, two types of viruses that were mainly responsible for human infections, namely HSV-1 and HCoV-229E, were used as models for enveloped DNA and RNA viruses, respectively.

**Figure 5:**
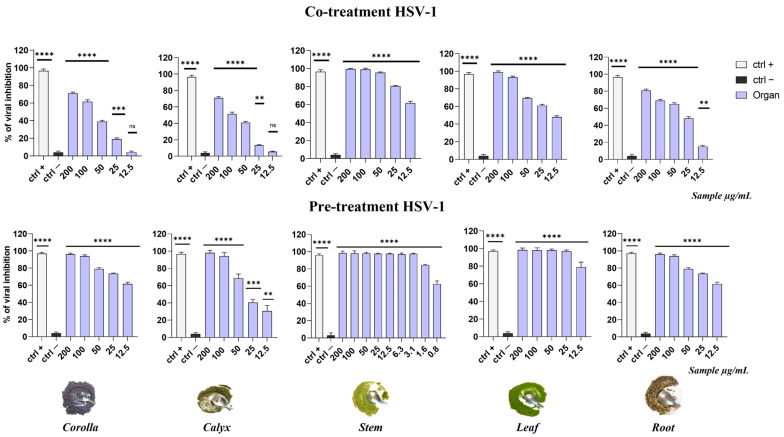
Comparison of antiviral activity against HSV-1 in co-treatment and pre-treatment assays. Rhamnolipid M15RL at 50 µg/mL [39] was used as positive control (ctrl+), while infected and non-treated cells were used as negative control (ctrl−). **** *p* < 0.0001; *** *p* = 0.0002; ** *p* = 0.0012; ns: non-significant.

As shown in Figure 5, all the extracts led to a general reduction in HSV-1 replication when incubated on cells at the same time of the viral infection (co-treatment assay) in a dose-dependent manner. In particular, among them, the stem extract displayed the best activity, and its lowest tested concentration (12.5 µg/mL) showed a viral inhibition of 60%. Afterwards, the leaf extracts exhibited an inhibition of 50% at the lowest concentrations while calyx and corolla were the least active extracts. In the pre-treatment assay, a general improvement of antiviral activity occurred for all the extracts. In particular, the stem extract showed the most remarkable antiviral effect, which, compared to that of the co-treatment assay, was increased, allowing the complete viral inhibition at 3.1 µg/mL.

**Figure 6:**
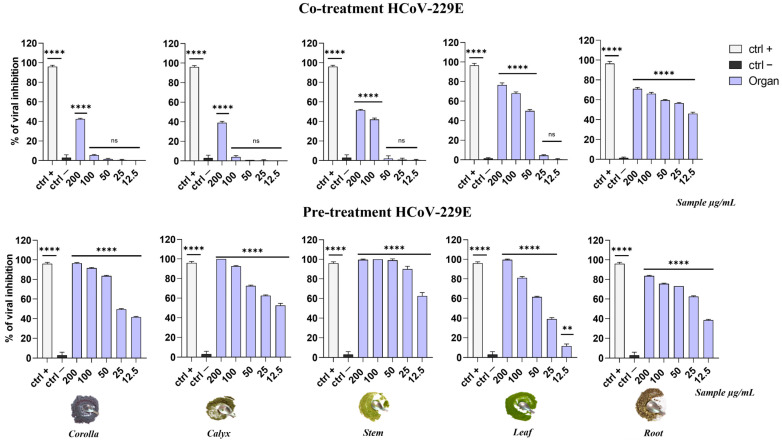
Comparison of antiviral activity against HCoV-229E in co-treatment and pre-treatment assays. Rhamnolipid M15RL at 50 µg/mL [39] was used as positive control (ctrl+), while infected and non-treated cells were used as negative control (ctrl−). **** *p* < 0.0001; ** *p* = 0.0060; ns: non-significant.

Studies performed on HCoV-229E displayed that the extracts showed a general trend comparable with HSV-1 results. In detail, as reported in Figure 6, in the co-treatment assay the extracts did not exhibit a significant activity with the exception of the roots extract, whose viral inhibition was around 60%.

Surprisingly, in the pre-treatment test, even fewer active extracts showed a great viral inhibition. The stem extract displayed the best activity among the others, with an IC_90_ equal to 30.3 µg/mL.

These promising preliminary results suggested a possible involvement of the extracts in an early and extracellular stage of the viral life cycle before the virus penetration. Then, we performed a cell pre-treatment and post-treatment assay against the same viruses. The results are shown in Supplementary Materials (Figure S2a,b). No significant inhibition was observed at any tested concentrations against both the viral models.

The data confirm that *L. austroapennina* extracts act directly on viral particles before entry in the host cell. Since the two selected viruses (HSV-1 and HCoV-229E) are both enveloped viral models, the extracts’ mode of actions were further investigated by performing a plaque reduction assay in the best condition of activity (pre-treatment) with a non-enveloped virus, namely PV-1.

**Figure 7:**
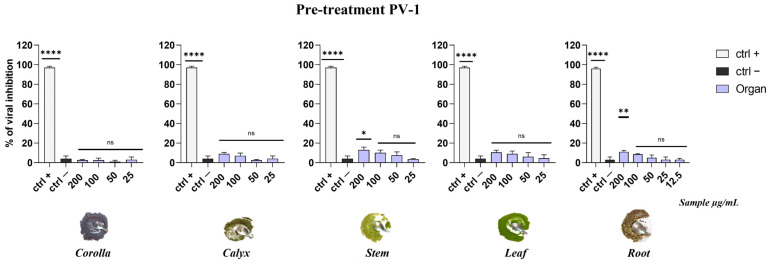
Antiviral activity against a non-enveloped virus. Data show that the extracts do not inhibit viral replication. Positive control (ctrl+) was pleconaril (2 µg/mL). Infected and non-treated cells were used as negative control (ctrl−). **** *p* < 0.0001; ** *p* = 0.0045; * *p* = 0.0424; ns: non-significant.

The graphs in Figure 7 demonstrated that the extracts were not able to inhibit a poliovirus infection, suggesting that their mode of action could be limited to the viral envelope. In general, the apolar extracts were most effective when incubated with HSV-1 and HCoV-229E upon addition to the target cells (co-treatment) or with the virus (pre-treatment) prior to the infection on the cell monolayer. On the contrary, no significant activity was detected when the extracts were added after HSV-1 and HCoV-229E infection (post-treatment) or when cells were first treated with the extracts and then infected (cell pre-treatment).

## 4. Discussion

The apolar extracts from *L. austroapennina* organs exhibited antiviral activity on both HSV-1 and HCoV-229E. The former is a double-stranded DNA, enveloped virus, responsible for labial herpes, and rarely constitutes the causative agent of more severe diseases, such as keratitis and encephalitis which are often fatal in immunocompromised people [40]. HSV infections are not eradicable; in fact, after primary infection, viruses enter the nerve cells, giving rise to lesions in the host and inducing the development of lifelong latent infection in sensory neurons. Fever, stress, or immunosuppression could reactivate and rapidly relapse the occurrence of symptoms [41]. The available treatments are based on several selective drugs which, however, has led to troublesome secondary effects and drug-resistant strains [42]. Thus, the development of new antiviral agents and the discovery of new sources of antiherpetic drugs are challenges. The promising activity of apolar extracts from *L. austroapennina* organs is in line with the data by Yucharoen et al. [43], who revealed that lavender dichloromethane extract, compared to sage and chamomile, was effective on HSV-1 at low doses during viral absorption and after viral absorption. A recent case report highlighted that the treatment with a botanical blend containing *L. officinalis* (10%), *Hypericum perforatum, Glycyrrhiza glabra*, *Melissa officinalis*, *Eleutherococcus senticosus*, and *Sarracenia* (mixed species) was able to reduce the oro-facial herpes symptoms (72 h) and the frequency of future outbreaks [44].

HCoV-229E infection has been also affected by *L. austroapennina* extracts. This enveloped virus has a single-stranded RNA genome and causes epidemic outbreaks during winters [45]. It belongs to the human-less pathogenic coronavirus group (HCoV-229E, HCoV-OC43, HCoV-NL63, and HCoV-HKU1) responsible for mild respiratory tract infections resembling the common cold. Instead, three other coronaviruses have posed a severe danger to human health in the last few years: the Middle East respiratory syndrome coronavirus (MERS-CoV), severe acute respiratory syndrome coronavirus (SARS-CoV), and the novel SARS-CoV-2 [46]. The last pandemic event highlights the need of new effective therapeutic options against coronavirus infections [47]. When a co-treatment assay was carried out using *L. austroapennina* apolar extracts, it was observed that the highest concentration of both corolla and calyx extracts exhibited a significant viral replicative inhibition, while stem and leaf extracts were active at 100 and 50 µg/mL, respectively, and the root sample exerted virus inhibition close to 50% at 12.5 µg/mL. The extracts appeared to be more effective when a pre-treatment experiment was carried out, with the only exception of the root sample, which showed a similar trend. The root extract appeared peculiarly enriched in docosyl ferulate (**20**). This compound was shown as a behaviourally active GABAA receptor complex (GABAAR) agonist [48], whose occurrence has not been reported in the *Lavandula* species.

Stem sample at 12.5 and 25 µg/mL induced viral inhibition of ~60% and ~90%, respectively. Although there are no data on the antiviral activity of the *Lavandula* species against HCoV-229E, there are various reports in relation to SARS-CoV-2 and avian influenza virus (H5N1), which share similar morphology (RNA-virus with envelope) and symptoms (respiratory disease). In this regard, essential oils are of interest as phytochemicals that are able to interfere with the enveloped viral lipid bilayer or inhibit specific processes in the replication cycle, preventing viral cellular shedding [46]. Recently, EOs of *Lavandula angustifolia* and *Salvia officinalis* were found to be active against H5N1 [17], and linalool and camphor—the main compounds of *L. angustifolia, L. stoechas,* and *L. × heterophylla* EOs—were shown to inhibit ACE-2 and LOX enzymes involved in the SARS-CoV-2 infection [49]. Clinical studies have shown that the oral administration of EOs from *Lavandula latifolia* reduce the symptoms of acute respiratory infections by viruses belonging to the *Coronaviridae* family. In fact, the treatment of patients with Tavipec^®^ capsules, providing 900 mg of EO from the flowering tops and stems of spike lavender, allowed the observation of an improvement in the symptoms of acute rhinosinusitis and a decrease in the symptoms of acute bronchitis versus a placebo [50].

The antiviral drugs can exploit different mechanisms such as the inhibition of virus attachment, entry and uncoating, or it can interfere with viral enzymes, such as polymerase and protease as well as reverse transcriptase and integrase [6]. Data herein collected suggested that *L. austroapennina* organs differently induce viral suppression acting in the early stages of a viral replication, through disruption of the pathogen envelope, which is derived from the host cell plasma membrane. In fact, fatty acids, which represent the most abundant component for almost all the extracts investigated, were capable of inducing the inactivation of some enveloped viruses [51,52]. Medium-chain saturated fatty acids (capric, lauric, and myristic acids) and unsaturated fatty acids such as oleic, linoleic, and palmitoleic acids, exhibited marked antiviral activity against enveloped vesicular stomatitis virus (VSV), HSV, and HIV [53]. Fatty acids and 1-monoglycerides have also been found to kill pathogens known to infect the mucosa and skin [54]. The most plausible mechanism of action was based on the ability of these compounds to enter the lipid membranes of the virus and to destroy its function, thanks to the intrinsic amphipathic and lipophilic properties [55]. Palmitic acid has also been described to have potent antiviral activity against HIV-1 and HIV-2 [56], and free fatty acids such as oleic and linoleic acid can inactivate enveloped viruses such as herpes and the flu. Furthermore, the exogenous supplementation of linoleic acid or arachidonic acid into infected cells suppressed the replication of HCoV-229E and the highly pathogenic MERS-CoV. This evidence suggests a positive outcome for the use of apolar mixtures from *L. austroapennina*. The effect of triterpenes cannot be excluded. Ursolic acid and oleanolic acid have been shown to prevent virus adsorption and invasion into host cells in the early stages and to inhibit the viral replication process after cell infection [57]. Oleanolic acid exerted antiviral activity against both normal strains of HSV-1 and against ACV-resistant strains (HSV-1/blue, HSV-1/106, and HSV-1/153), and it has been suggested that it may exert its anti-HSV-1 activity through the deregulation of UL8, a component of the virus helicase–primase complex [58,59]. Both ursolic and oleanolic acids were found to be potential inhibitors against the major protease (Mpro) of SARS-CoV-2, using integrated molecular modeling approaches, like acting and controlling viral replication [60]. Ursolic acid has already been extensively investigated, by inhibiting HSV-1 and HSV-2 at 14.5 μg/mL within 2–5 h after infection [61]. The efficacy data of ursolic acid even allowed the drawing of an opinion report on its effect against SARS-CoV-2, which was able to mitigate post-COVID-19 complications such as pulmonary fibrosis [62].

## 5. Conclusions

This study underlines how much knowledge of a territory’s medicinal plant heritage is a harbinger of promising discoveries in the health field. On the other hand, the definition of the chemical profile of a plant extract is mandatory to obtain an optimal recovery and exploitation of the bioactive components. The chromatographic analyses in high-resolution mass spectrometry showed that the extracts were discriminable on the basis of the relative amount of the identified fatty acids and triterpenes. In particular, oleanolic acid was the most abundant triterpene in the stem extract, and its content was higher than that of ursolic acid in the leaf, whereas a comparable content of ursolic acid and oleanolic acid was in the corolla. The calyx extract was distinguished by the palmitic acid content. The extracts were evaluated for cytotoxicity on the Vero cell line and analyzed for their antiviral capacity against HSV-1 and HCoV-229E infection. The stem extract showed an interesting antiviral efficacy in the pre-treatment assay, and its dose equal to 3.1 µg/mL completely inhibited HSV-1 replication. Analogously, against HCoV-229E, the stem extract displayed a strong activity exhibiting an IC_90_ value equal to 30.3 µg/mL. This study underlines how much knowledge of a territory’s medicinal plant heritage is a harbinger of promising discoveries in the health field. On the other hand, the definition of the chemical profile of a plant extract is mandatory to obtain an optimal recovery and exploitation of the bioactive components.

## Figures and Tables

**Table 1:** UHPLC-ESI-QqToF/MS and MS/MS data for tentatively identified compounds in apolar mixtures from *L. austroapennina* organs.

Peak n.	RT (min)	Tentative Assignment	Formula	[M-H]^−^ Found (*m*/*z*)	Error (ppm)	RDB	MS/MS Fragment Ions (*m*/*z*)
**1**	0.672	Diterpene (e.g., rosmaridiphenol)	C_20_H_28_O_3_	315.1961	−1.5	7	315.1962; 283.1694; 227.1076
**2**	0.682	Diterpene (e.g., rosmanol)	C_20_H_26_O_5_	345.1707	−1.0	8	345.1695; 330.1474; 300.1360
**3**	1.140	dihydroxy-octadecadienoic acid	C_18_H_32_O_4_	311.2225	−0.9	3	311.2217; 293.2119; 275.1970
**4**	1.569	9-oxooctadeca-10,12-dienoic acid	C_18_H_30_O_3_	293.2117	0.6	4	293.2117; 275.2007; 235.1689; 183.1388; 171.1024
**5**	1.728	Diterpene derivative (e.g., rosmaquinone B)	C_21_H_26_O_5_	357.1712	1.3	9	357.1709; 311.1646; 296.1413; 241.0861
**6**	1.782	9-oxooctadeca-10,12,15-trienoic acid	C_18_H_28_O_3_	291.1966	1.8	5	291.1966; 273.1854; 247.2058; 181.1254
**7**	1.877	Diterpene (e.g., carnosic acid)	C_20_H_28_O_4_	331.1919	1.3	7	331.1912; 303.1952
**8**	1.993	trihydroxy urs-12-en-28-oic acid	C_30_H_48_O_5_	487.3435	1.2	7	487.3442; 469.3331; 443.3543; 425.3430; 409.3113
**9**	2.535	dioxo-hydroxyurs-12-ene-28-oic acid	C_30_H_44_O_5_	483.3117	0.2	7	483.3118; 465.3017; 439.3200; 421.3106; 379.2992
**10**	2.903	3-oxo-hydroxyurs-12-en-28-oic acid	C_30_H_46_O_4_	469.3330	1.4	8	469.3344; 451.3223; 407.3311
**11**	3.281	hydroxyoctadec-12-enoic acid	C_18_H_34_O_3_	297.2446	3.6	2	297.2454; 279.2341; 155.1085
**12**	4.544	hydroxy-3-oxo-ursa-1,12-dien-28-oic acid	C_30_H_44_O_4_	467.3175	1.7	9	467.3184; 449.3077
**13**	5.166	linolenic acid	C_18_H_30_O_2_	277.2178	1.7	9	277.2176
**14**	5.438	oleanolic acid	C_30_H_48_O_3_	455.3539	1.8	7	455.3538
**15**	5.671	ursolic acid	C_30_H_48_O_3_	455.3536	1.2	7	455.3543
**16**	5.909	linoleic acid	C_18_H_32_O_2_	279.2334	1.6	3	279.2335
**17**	6.076	dehydroursolic acid	C_30_H_46_O_3_	453.3381	1.5	8	453.3392; 407.3312
**18**	6.471	palmitic acid	C_16_H_32_O_2_	255.2334	1.4	1	255.2331; 219.8351
**19**	6.723	oleic acid	C_18_H_34_O_2_	281.2489	1.1	2	281.2491
**20**	7.889	docosyl ferulate	C_32_H_54_O_4_	501.3962	2.5	6	501.3962; 486.3723; 193.0508; 177.0196; 133.0299

## Data Availability

The data presented in this study are available on request from the corresponding author. The authors can confirm that all relevant data are included in the article.

## Supplementary Materials

The following supporting information can be downloaded at: https://www.mdpi.com/article/10.3390/v15081648/s1, Figure S1: TOF-MS/MS spectra and proposed chemical structure of compounds: (A) 1; (B) 2; (C) 5; (D) 7. Figure S2: TOF-MS/MS spectra and hypothesized structures of compounds: (A) 3; (B) 4; (C) 6; (D) 11; (E) 13; (F) 16; (G) 18; (H) 19. Figure S3: TOF-MS/MS spectra and hypothesized structures of compounds: (A) 14; (B) 15; (C) 17; (D) 9; (E) 10; (F) 12. Figure S4: (A) TOF-MS/MS spectrum and (B) proposed fragmentation pattern of compound 8. Theoretical *m*/*z* values are reported below each structure. Figure S5: comparison of antiviral activity against HSV-1 in post-infection and cell pre-treatment assay. Figure S6: comparison of antiviral activity against HCoV-229E in post-infection and cell pre-treatment assay.
