# Supplementary material for: Lavandula austroapennina: Assessment of the Antiviral Activity of Lipophilic Extracts from Its Organs
Source: Viruses. 2023 Jul 28;15(8):1648. doi: 10.3390/v15081648 (PMC10457779; doi:10.3390/v15081648)
Supplement: Supplementary file 1 [file viruses-15-01648-s001.zip › viruses-2495287-supplementary.pdf]

Supplementary Materials

Article

Lavandula austroapennina: Assessment of the Antiviral Activity of Lipophilic Extracts from its Organs

Chianese Annalisa [1], Claudia Gravina [2], Mariavittoria Morone [1], Marialuisa Formato [2], Annalisa Ambrosino [1], Rosa Giugliano [1], Bianca Maria Nastri [1], Carla Zannella [1], Assunta Esposito [2], Anna De Filippis [1], Simona Piccolella [2], Massimiliano Galdiero [1,*,] Severina Pacifico [2,*]

[1] Department of Experimental Medicine, University of Campania "Luigi Vanvitelli", 80138 Naples, Italy
[2] Department of Environmental, Biological and Pharmaceutical Sciences and Technologies, University of Campania 'Luigi Vanvitelli', 81100 Caserta, Italy

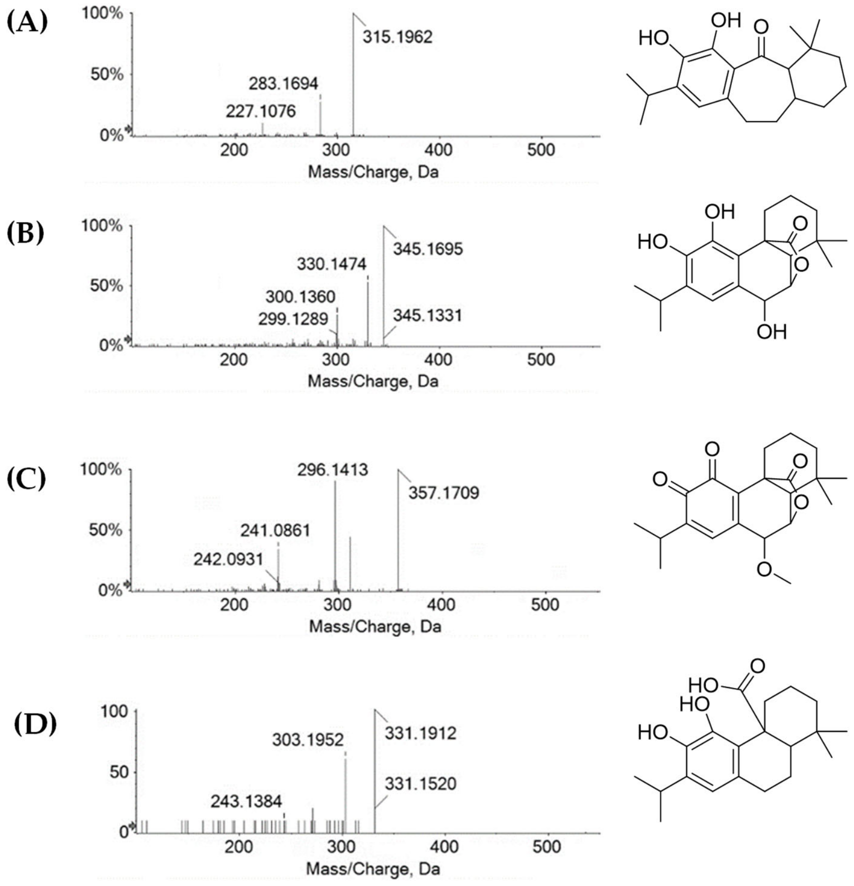

Figure S1. TOF-MS/MS spectra and proposed chemical structure of compounds: (**A**) 1; (**B**) 2; (**C**) 5; (**D**) 7.

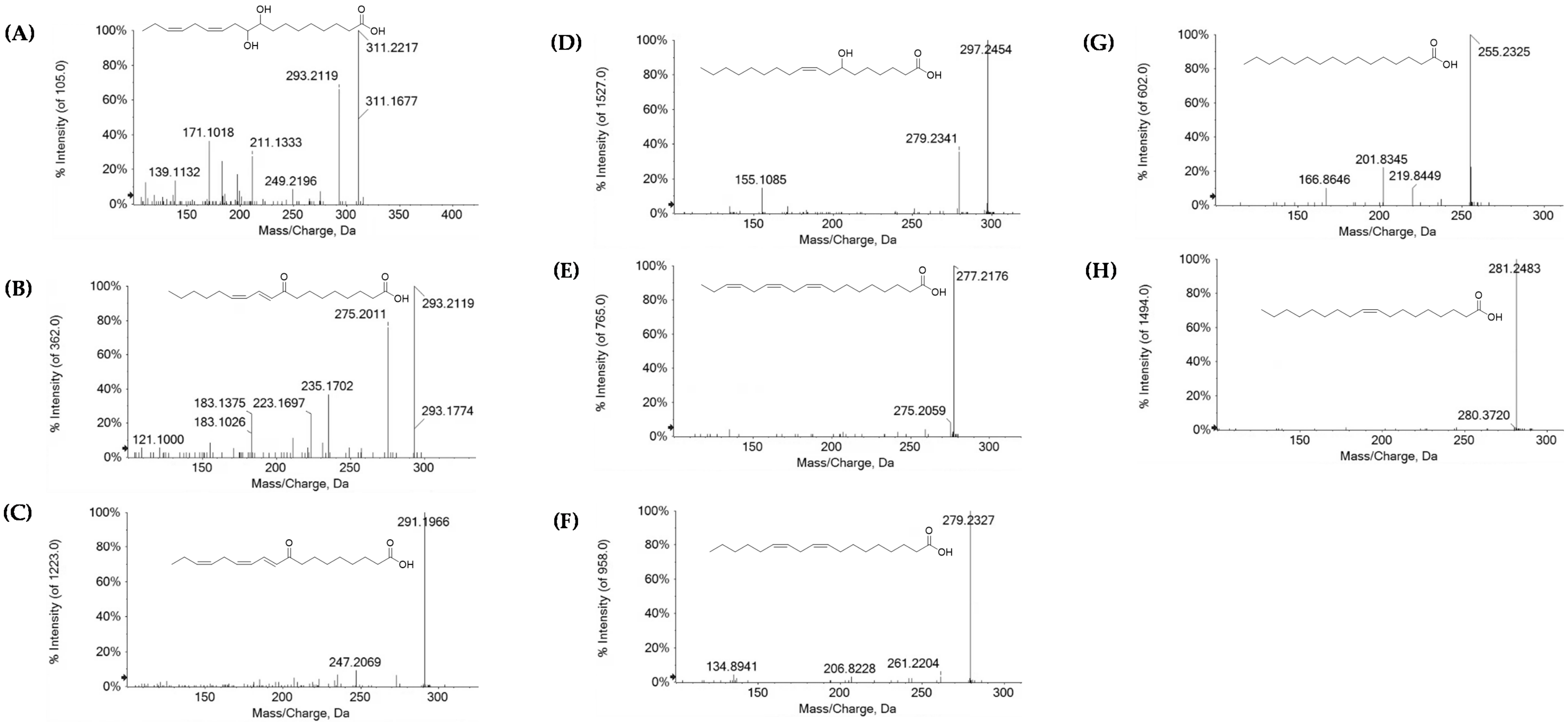

Figure S2. TOF-MS/MS spectra and hypothesized structures of compounds: (**A**) 3; (**B**) 4; (**C**) 6; (**D**) 11; (**E**) 13; (**F**) 16; (**G**) 18; (**H**) 19.

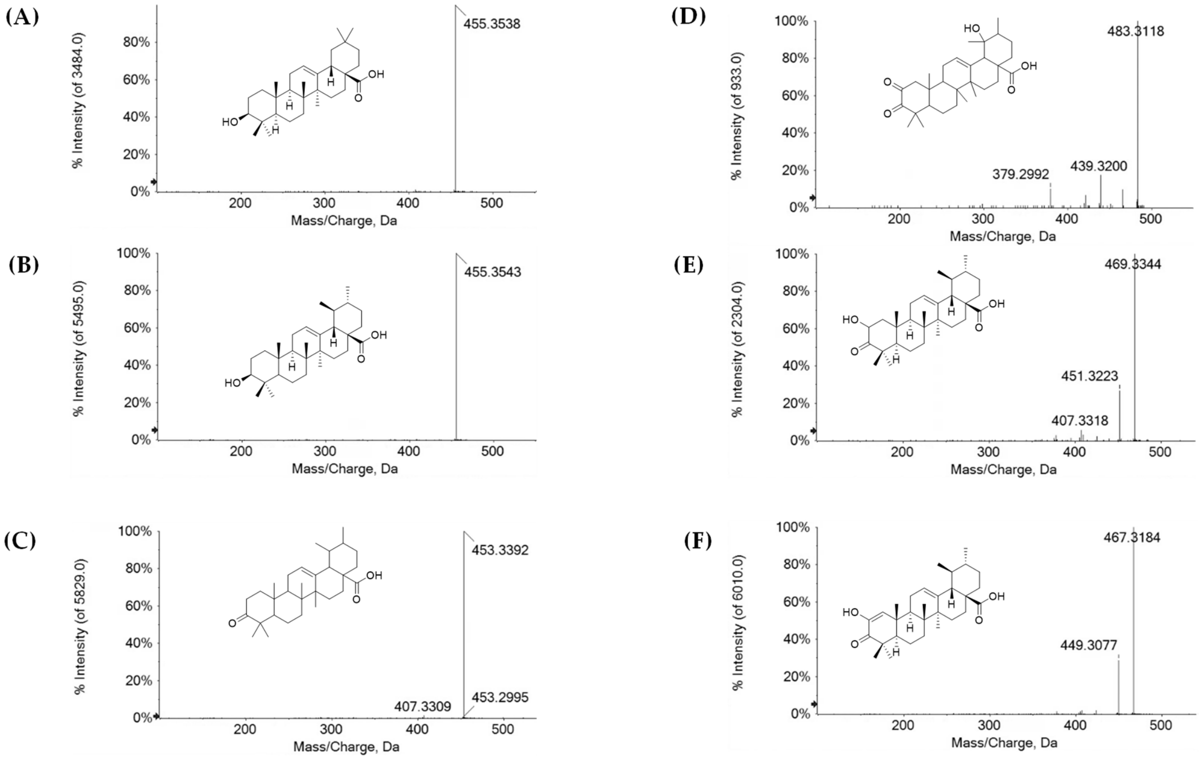

Figure S3. TOF-MS/MS spectra and hypothesized structures of compounds: (**A**) 14; (**B**) 15; (**C**) 17; (**D**) 9; (**E**) 10; (**F**) 12.

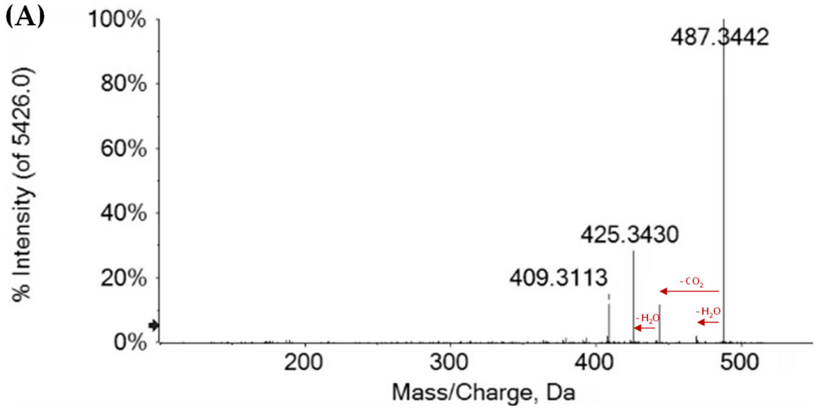

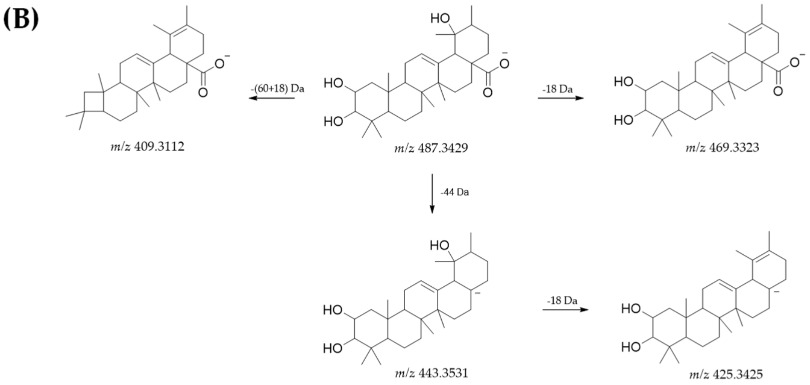

Figure S4. (**A**) TOF-MS/MS spectrum and (**B**) proposed fragmentation pattern of compound **8**. Theoretical *m*/*z* values are reported below each structure.

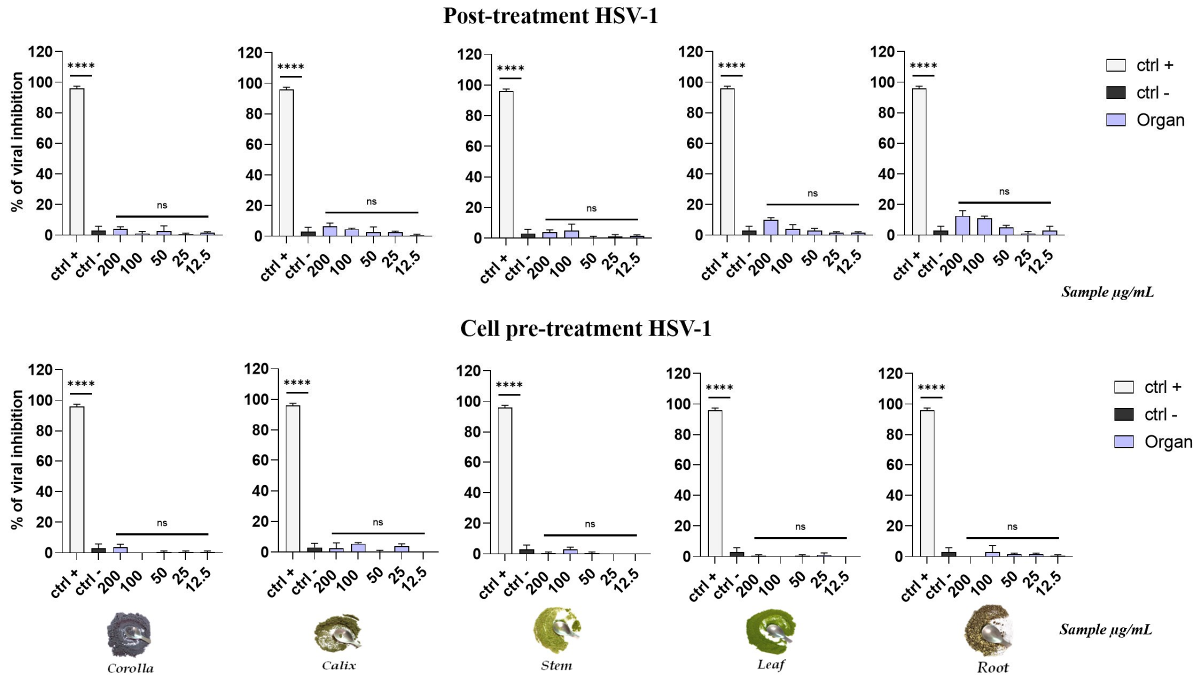

Figure S5. Comparison of antiviral activity against HSV-1 in post infection and cell pre-treatment assay.

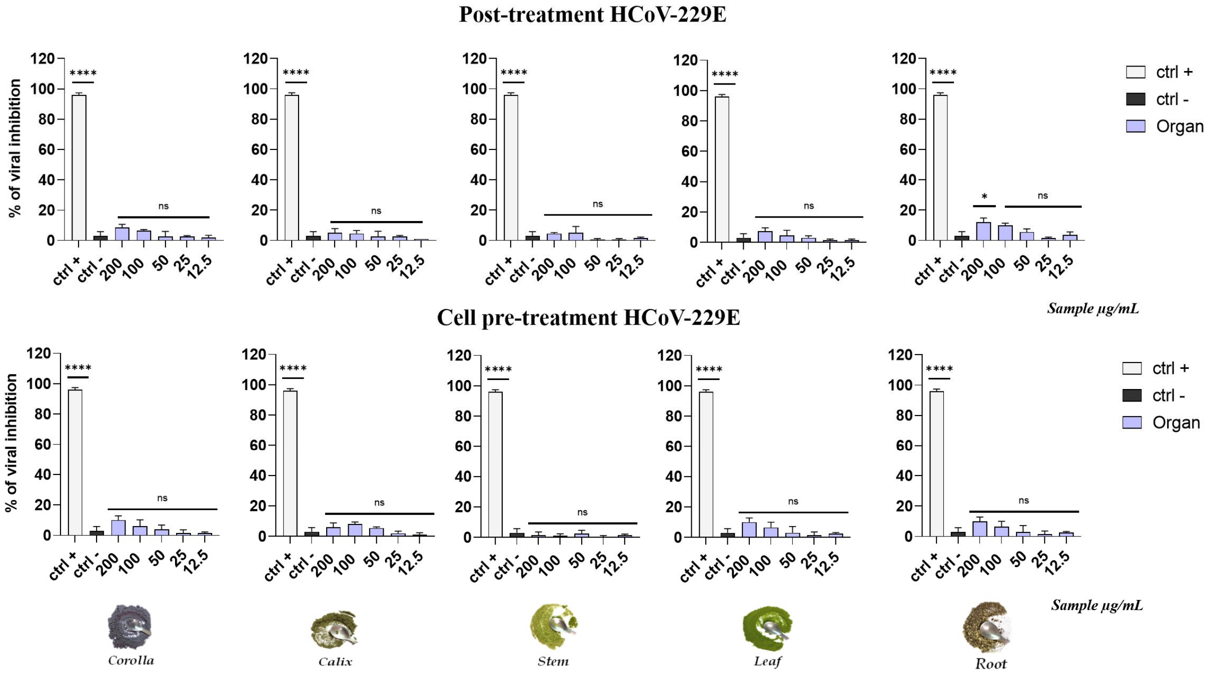

Figure S6. Comparison of antiviral activity against HCoV-229E in post infection and cell pre-treatment assay.
